# PReS-FINAL-2342: Anti-TNFALPHA therapy targeys PKB/C-AKT induced resistance of effector cells to suppression in juvenile arthritis

**DOI:** 10.1186/1546-0096-11-S2-P332

**Published:** 2013-12-05

**Authors:** E Wehrens, S Vastert, G Mijnheer, J Meerding, M Klein, N Wulffraat, B Prakken, F van Wijk

**Affiliations:** 1 Pediatric Immunology, Wilhelmina Childrens Hospital, University Medical Center, Utrecht, Netherlands

## Introduction

Resistance of effector T cells (Teff) to suppression contributes to disturbed immune regulation in autoimmune disease. Targeting this unresponsiveness to suppression might therefore have beneficial effects in autoimmune inflammation. In juvenile idiopathic arthritis (JIA) we have recently shown that Teff from inflamed joints are refractory to suppression, which was associated with enhanced PKB/c-akt activation in these cells.

## Objectives

To investigate whether anti-IL-6 and anti-TNFα target unresponsiveness of Teff to suppression in patients with JIA.

## Methods

Resistant Teff from the inflamed joints of JIA patients were cultured in the presence of etanercept or anti-IL-6 *in vitro *and PKB/c-akt activation and responsiveness to suppression was measured. In addition, *in vivo *effects of TNFα blockade were investigated using peripheral blood samples of patient before and after start of etanercept therapy.

## Results

*In vitro *treatment of synovial fluid Teff with anti-IL-6 led to improved Treg mediated suppression of cell proliferation in some, but not all patients. Blocking TNFα with etanercept however clearly enhanced suppression in all samples analyzed. In the presence of etanercept PKB/c-akt activation of Teff was reduced and Teff became more susceptible to TGFβ-mediated suppression, indicating that anti-TNFα directly targets resistant Teff.

## Conclusion

This study is the first to show resistance of Teff to suppression as a target of anti-TNFα therapy in arthritis, resulting in improved regulation of inflammatory effector cells.

## Disclosure of interest

E. Wehrens: None Declared, S. Vastert Consultant for: novartis < 1000 euros, G. Mijnheer: None Declared, J. Meerding: None Declared, M. Klein: None Declared, N. Wulffraat Grant/Research Support from: Abbvie, Roche, Consultant for: Novartis, Pfizer, B. Prakken: None Declared, F. van Wijk: None Declared.

